# Nitrogen addition regulates the effects of variation in precipitation patterns on plant biomass formation and allocation in a *Leymus chinensis* grassland of northeast China

**DOI:** 10.3389/fpls.2023.1323766

**Published:** 2024-01-12

**Authors:** Jianli Ren, Chengliang Wang, Qiaoxin Wang, Wenzheng Song, Wei Sun

**Affiliations:** ^1^ Institute of Grassland Science, Key Laboratory of Vegetation Ecology of the Ministry of Education, Jilin Songnen Grassland Ecosystem National Observation and Research Station, Northeast Normal University, Changchun, Jilin, China; ^2^ Institute of Resources and Ecology, Yili Normal University, Yining, Xinjiang, China; ^3^ School of Resources and Environment, Yili Normal University, Yining, Xinjiang, China; ^4^ College of Tourism, Resources and Environment, Zaozhuang University, Zaozhuang, China; ^5^ State Environmental Protection Key Laboratory of Wetland Ecology and Vegetation Restoration, Northeast Normal University, Changchun, Jilin, China

**Keywords:** precipitation amount, precipitation frequency, nitrogen addition, plant biomass, biomass allocation, meadow steppe, *Leymus chinensis*

## Abstract

Global warming is predicted to change precipitation amount and reduce precipitation frequency, which may alter grassland primary productivity and biomass allocation, especially when interact with other global change factors, such as nitrogen deposition. The interactive effects of changes in precipitation amount and nitrogen addition on productivity and biomass allocation are extensively studied; however, how these effects may be regulated by the predicted reduction in precipitation frequency remain largely unknown. Using a mesocosm experiment, we investigated responses of primary productivity and biomass allocation to the manipulated changes in precipitation amount (PA: 150 mm, 300 mm, 450 mm), precipitation frequency (PF: medium and low), and nitrogen addition (NA: 0 and 10 g N m^−2^ yr^−1^) in a *Leymus chinensis* grassland. We detected significant effects of the PA, PF and NA treatments on both aboveground biomass (AGB) and belowground biomass (BGB); but the interactive effects were only significant between the PA and NA on AGB. Both AGB and BGB increased with an increment in precipitation amount and nitrogen addition; the reduction in PF decreased AGB, but increased BGB. The reduced PF treatment induced an enhancement in the variation of soil moisture, which subsequently affected photosynthesis and biomass formation. Overall, there were mismatches in the above- and belowground biomass responses to changes in precipitation regime. Our results suggest the predicted changes in precipitation regime, including precipitation amount and frequency, is likely to alter primary productivity and biomass allocation, especially when interact with nitrogen deposition. Therefore, predicting the influence of global changes on grassland structure and functions requires the consideration of interactions among multiple global change factors.

## 1 Introduction

Continued global warming has altered worldwide precipitation patterns, including precipitation amount, precipitation intensity, precipitation interval, and precipitation timing (Bodelier, 2011; Fay et al., 2015; IPCC, 2021; Gao et al., 2023). Previous studies have shown that changes in the precipitation amount and frequency are likely to alter soil wet and dry cycles, which in turn affects key ecosystem functions, such as primary productivity (Nielsen and Ball, 2015; Wu et al., 2021; Du et al., 2023). In addition to variations in precipitation patterns, terrestrial ecosystems are facing human activity-associated enhancement in nitrogen deposition (Gruber and Galloway, 2008). As a key limiting factor of primary productivity in most terrestrial ecosystems, including grasslands, nitrogen deposition-related nutrient inputs are likely to alter responses of vegetation composition and ecosystem functions to variations in precipitation patterns.

Grasslands often occur in arid and semi-arid regions where ecosystem functions, such as plant biomass accumulation and allocation, are highly sensitive to changes in precipitation patterns (mainly precipitation amount and precipitation frequency) (Gilbert et al., 2020; Ma et al., 2022). In general, carbon assimilation rate and aboveground biomass accumulation in grasslands are often positively associated with precipitation amount (Yu et al., 2022; Yang et al., 2023b); however, the root-to-shoot ratio often negatively correlated with precipitation amount (Wang et al., 2018; Qi et al., 2019). In addition to influencing primary productivity and allocation, changes in precipitation amount could also profoundly alter plant community structure and composition. For instance, some short-term manipulative experiments have demonstrated that elevated precipitation notably enhanced plant diversity (Zavaleta et al., 2003; Yang et al., 2011). On top of changes in precipitation amount, variation in precipitation frequency may alleviate or amplify the effects of changes in precipitation amount on ecosystem functions depending on dominant species characteristics and soil texture (Shang et al., 2019; Zhang and Zhao, 2022). Low precipitation frequency causes topsoil to undergo a more substantial wet–dry cycle and may result in a reduction in nitrogen content (more leaching and denitrification), which may subsequently impair plant growth (Feng et al., 2023; Wang et al., 2023). As an adaptation to low precipitation frequency, plants are likely to allocate more biomass to deeper soil where the soil moisture environment is more stable (Knapp et al., 2008; Wilcox et al., 2015; Post and Knapp, 2020). Apparently, the effects of variation in precipitation frequency on plant biomass formation and allocation likely depend on precipitation amount, but these interactive effects are seldom studied.

In addition to water, nitrogen availability is another key limiting factor for grassland primary productivity. Indeed, a few studies have reported strong interactions between nitrogen addition and precipitation variation on vegetation structure and ecosystem functions (Xu et al., 2012a; Lü et al., 2018). Nitrogen addition often leads to greater leaf photosynthetic rate and higher leaf area, which subsequently increase transpiration and water consumption rate (Liang et al., 2020; Wang et al., 2021; Yang et al., 2023a). Nitrogen addition is likely to further reduce belowground biomass allocation due to alleviation of soil nutrient limitation (Chen et al., 2018; Yan et al., 2022). However, the results of some studies have shown that nitrogen addition mitigates the negative effects of drought on plants (Grossiord et al., 2018; Shi et al., 2022). In another way, long-term nitrogen addition is likely to change vegetation composition and adaptability to variations in precipitation patterns (Yang et al., 2011; Smith et al., 2016). Indeed, some studies have found that the effects of nitrogen on plant community are strongly mediated by water availability in the grasslands and that increased precipitation often reduces the negative effects of long-term nitrogen addition in the contribution of forbs to aboveground productivity (Xu et al., 2012b; Li et al., 2019b). To date, it is not clear how nitrogen addition regulates the effects of changes in precipitation amount and precipitation frequency on biomass formation and allocation.

Using soil columns collected from a *Leymus chinensis* meadow in northeast China, we conducted a mesocosm experiment using manipulated changes in precipitation amount and frequency under the conditions of with and without nitrogen addition (10 g N m^−2^ year^−1^). We measured soil water content, plant aboveground biomass (AGB), belowground biomass (BGB), and leaf gas exchange of the dominant plant. We hypothesized that i) AGB and BGB are likely to increase with increasing precipitation amount and nitrogen addition and precipitation frequency reduction may decrease AGB but increase BGB; ii) reducing precipitation frequency leads to greater fluctuation in soil moisture, which is likely to increase BGB allocation to deeper soil layer; and iii) nitrogen addition is likely to enhance the contribution of non-dominant species to total aboveground biomass especially when precipitation amount was enhanced.

## 2 Materials and methods

### 2.1 Study site

This study was conducted at the Songnen Grassland Ecosystem National Observation and Research Station (44°34′25″N, 123°31′6″E), which was located in western Jilin province, northeast China. The study area has a temperate semi-arid continental climate, with a mean annual air temperature of 6.4°C (Meng et al., 2021a), average annual precipitation of 445 mm, and over 80% of precipitation occurring during the growing season (from May to September) (Li et al., 2019a). The zonal soil at the study site is classified as Salic Solonetz (World Reference Base for Soil Resources) or an Aqui-Alkalic Halosol (Chinese soil classification) with a pH of 8.0–9.0 (Gao et al., 2023). Soil texture (28.9% clay, 40.1% silt, and 31.0% sand) corresponds to clay loam soil (Shi et al., 2021). The bulk density is 1.44 g/cm^3^, and the field capacity is approximately 0.26 g/g (Meng et al., 2021b). Soil total carbon, organic carbon, and total N concentrations are 7.22 ± 0.05 mg/g, 6.58 ± 0.11 mg/g, and 0.74 ± 0.01 mg/g, respectively (Shi et al., 2021). The vegetation is dominated by the C_3_ rhizomatous perennial grass *L. chinensis*; other perennial grasses, such as *Phragmites australis* and *Kalimeris integrifolia*, and annual plants, such as *Chloris virgata*, are abundant (Wang et al., 2018).

### 2.2 Experimental design

In 2010, one hectare (100 m × 100 m) of grassland area (historically used for light grazing and hay production) was fenced to exclude disturbances. Five blocks (each had an area of 20 m × 10 m) were randomly laid out with at least 1-m space between the blocks. Each block was divided into two plots and randomly assigned to the control treatment (N0, with no nitrogen addition) and nitrogen addition treatment (N10, 10 g N m^−2^ year^−1^). For the grassland ecosystems of northern China, the thresholds for plant composition and biomass response to nitrogen were approximately 10 g N m^−2^ year^−1^ (Bai et al., 2010; Wang et al., 2019a). For the nitrogen addition treatment, urea was used as a fertilizer, and 5 g N m^−2^ was applied uniformly in May and early July from 2010 to 2019.

In October 2019, soil columns (diameter, 33 cm; height, 40 cm) were collected using a self-made soil column collector in the N0 and N10 plots. The collected soil columns (with vegetation) were placed in cylindrical PVC tubes (diameter, 33 cm; height, 45 cm) sealed at the bottom to form mesocosms (**Supplementary Figure S1**). The mesocosms were transported to the Jilin Songnen Grassland Ecosystem National Observatory and Research Station (15 km away from the sampling site) for the rainfall manipulation experiment. The mesocosms were placed in a rain shelter and arranged as five blocks, with each block having 12 mesocosms (6 N0 and 6 N10) (**Supplementary Figure S2**). The mesocosms were buried 40 cm into the ground to reduce environmental disturbance. After the placement, the mesocosms were manually watered (equal to 20 mm rainfall) to restore soil water status. From October 2019 to April 2020, the mesocosms received natural precipitation.

On April 30, 2020, the rain shelter was installed and used to isolate rainfall. A three-factor experimental design was used for this experiment: precipitation amount, precipitation frequency, and nitrogen addition. The manipulation experiment was conducted from May 1, 2020, to August 31, 2020. The precipitation amount and frequency settings were referenced from the Heisler-White study (Heisler-White et al., 2009). Daily precipitation data from 1968 to 2017 were collected through the China Meteorological Data Network (http://data.cma.cn). Based on the data of precipitation amount and frequency in the experimental area from May to August of 1968–2017, three levels of precipitation amount (PA) were set: medium precipitation amount (MPA; 300 mm), low precipitation amount (LPA; 150 mm), and high precipitation amount (HPA; 450 mm). The medium precipitation amount was equal to the long-term average precipitation from May to August (1968–2017) and served as the control in the present study. Two levels for precipitation frequency (PF) were set: medium precipitation frequency (MPF; four precipitation events per month) and low precipitation frequency (LPF; two precipitation events per month). To simulate the seasonal distribution of historical precipitation, the precipitation amount was assigned to each month based on the proportion of the monthly distribution of precipitation data for the experimental area from 1968 to 2017 (**Table 1**). For each precipitation event, a hand sprayer was used to add water to each mesocosm. See **Supplementary Figure S3** for dates of the imposed precipitation events. In total, there were 12 treatment combinations among precipitation amount, precipitation frequency, and nitrogen addition (continuation of aforementioned field treatments). This experiment ran from October 1, 2019, to September 1, 2020; see **Supplementary Figure S4** for dates of the experimental treatment and key parameter acquiring.

**Table 1:** Monthly distribution of the imposed three precipitation amount treatments.

Precipitation	May	June	July	August	Total precipitation
High precipitation amount (mm)	53.19	95.29	175.39	126.13	450
Medium precipitation amount (mm)	35.46	63.52	116.93	84.09	300
Low precipitation amount (mm)	17.73	31.76	58.46	42.05	150

### 2.3 Soil water content

Soil water content (0–10 cm) was measured using a soil moisture meter (TDR, TRIME-PICO32 IMKO, Ettlingen, Germany) on July 1, July 3, July 5, July 7, July 9, and July 11. The measuring dates covered one precipitation cycle (the fifth cycle) for the low precipitation frequency treatment and two precipitation cycles (the eighth and ninth cycles) for the medium precipitation frequency treatment. For each treatment combination, soil water content (SWC) was measured at five replications.

### 2.4 Leaf gas exchange

Leaf gas exchanges of *L. chinensis* were measured on the same dates for soil water content measurements. For each mesocosm, the uppermost fully expanded leaves were selected and marked for leaf gas exchange measurements. On each measuring date, leaf gas exchange parameters including net assimilation rate (*A*) and stomatal conductance were measured between 9:00 a.m. and 11:00 a.m. using a LI-6400 portable photosynthesis system (LiCOR Inc., Lincoln, NE, USA). The environmental parameters in the leaf chamber were set as follows: light intensity of 2,000 μmol m^−2^ s^−1^, air temperature of 25°C, and CO_2_ concentration of 400 μmol/mol. Leaf gas exchange measurements were repeated five times for each treatment combination on each measuring date.

### 2.5 Plant community composition and biomass

On September 1, 2020, all aboveground plants in the mesocosms were collected and separated by species. All plant species in the experiment were divided into two functional groups: dominant species (*L. chinensis*) and non-dominant species (graminoids: *P. australis*, *C. virgata*, and *Calamagrostis epigeios*; legumes: *Lespedeza davurica* and *Medicago ruthenica*; and forbs: *Potentilla flagellaris* and *K. integrifolia*) (Wang et al., 2018; Song et al., 2022). After plant biomass harvesting, soil columns were excavated and divided into two layers (0–10 cm and 10–40 cm), and the roots were collected by washing them out of the soil columns. The obtained aboveground and belowground plant samples were oven-dried at 105°C for 30 min and then at 65°C to a constant weight. The root-to-shoot ratio (R/S) was calculated as the ratio of BGB (0–40 cm) to AGB.

### 2.6 Statistical analyses

Linear mixed-effects models (LMMs) were used, with precipitation patterns and nitrogen treatments as fixed factors and blocks as random factors, to assess effects of the precipitation amount, precipitation frequency, nitrogen addition treatments and their interactions on AGB, BGB, root-to-shoot ratio, BGB (0–10 cm), BGB (10–40 cm), BGB (0–10 cm)/BGB, AGB of dominant species, AGB of non-dominant species, response ratio of aboveground biomass, and *A* of dominant species. Linear regression analysis was used to assess correlations between *A* and SWC and between AGB and *A*. The magnitude of the treatment effect was quantified as the response ratio, calculated as response ratio (%) = (T − C)/C × 100, where T is the value of a specific treatment and C is the value under the medium precipitation, medium precipitation frequency, and unfertilized conditions. All analyses were carried out using R v.4.1.2 software. The graphs used in this experiment were plotted using R v.4.1.2 software.

## 3 Results

### 3.1 Soil water content

There were significant PA treatment effects on 0–10-cm SWC in both fertilized and unfertilized mesocosms (**Supplementary Figure S5**). The nitrogen addition treatment significantly enhanced the SWC. In general, the LPF treatment had a higher magnitude of variation in SWC than the MPF treatment. Moreover, the PF reduction treatment-induced variation in SWC within a watering cycle tends to increase with increasing precipitation amount (**Supplementary Figure S5**).

### 3.2 Total plant biomass

**Figure 1:**
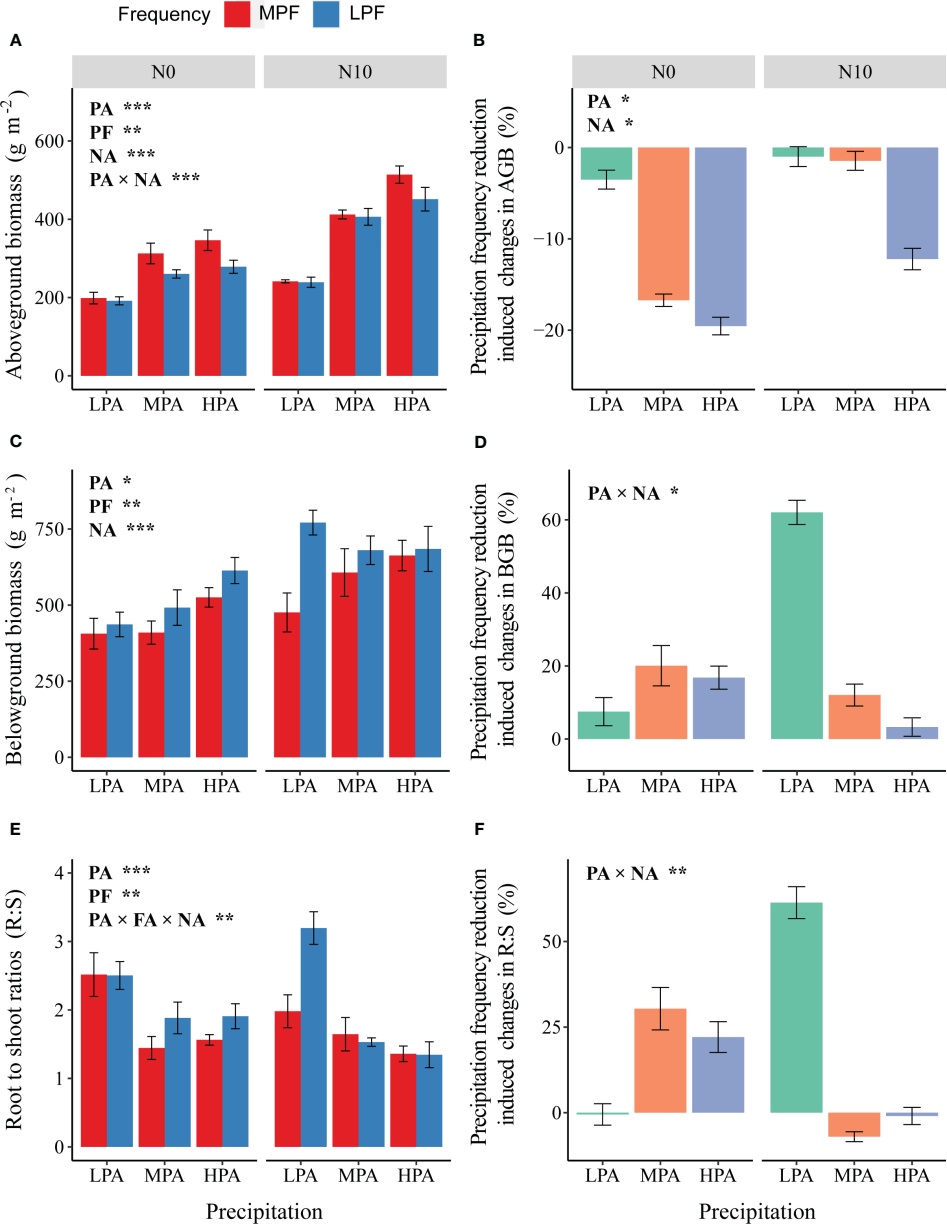
Effects of changes in precipitation pattern and nitrogen addition on aboveground biomass (**A**; AGB), belowground biomass (**C**; BGB), and root-to-shoot ratio (**E**; R:S). Differences in precipitation frequency reduction-induced changes in AGB **(B)**, BGB **(D)**, and R:S **F** among the precipitation and nitrogen addition treatments. The imposed treatments: precipitation amount (PA), low precipitation amount (LPA), medium precipitation amount (MPA), high precipitation amount (HPA), precipitation frequency (PF), low precipitation frequency (LPF), medium precipitation frequency (MPF), nitrogen addition (NA), nitrogen addition of 0 g N m^−2^ year^−1^ (N0), and nitrogen addition of 10 g N m^−2^ year^−1^ (N10). Data are presented as mean value ± 1 SE. Asterisks indicate the main treatment effects (*p < 0.05; **p < 0.01; ***p < 0.001).

For AGB, we detected significant effects of PA, PF, and nitrogen addition (NA), as well as between PA and NA (**Figure 1A**). In general, AGB increased with the nitrogen addition and increment in precipitation amount; however, the precipitation frequency reduction resulted in a decline in AGB (**Figure 1A**). The precipitation frequency reduction-induced changes in AGB were significantly affected by the PA and NA treatments. The precipitation frequency reduction-induced changes in AGB tended to increase with the increase in precipitation amount (**Figure 1B**). Moreover, the precipitation frequency reduction-induced changes in AGB were much less in the fertilized mesocosms (**Figure 1B**).

The PA, PF, and NA treatments had significant effects on BGB. The nitrogen addition, enhancement in precipitation amount, and reduction in precipitation frequency resulted in an increase in BGB (**Figure 1C**). For precipitation frequency reduction-induced changes in BGB, we only detected significant interactive effects between the PA and NA treatments (**Figure 1D**). For the root-to-shoot (R:S) ratio, we detected significant effects of the PA and PF treatments, as well as interactions among the PA, PF, and NA treatments (**Figure 1E**). In general, the R:S ratio decreased with the increase in precipitation amount, but it increased with the reduction of precipitation frequency (**Figure 1E**).

### 3.3 Belowground biomass allocation

**Figure 2:**
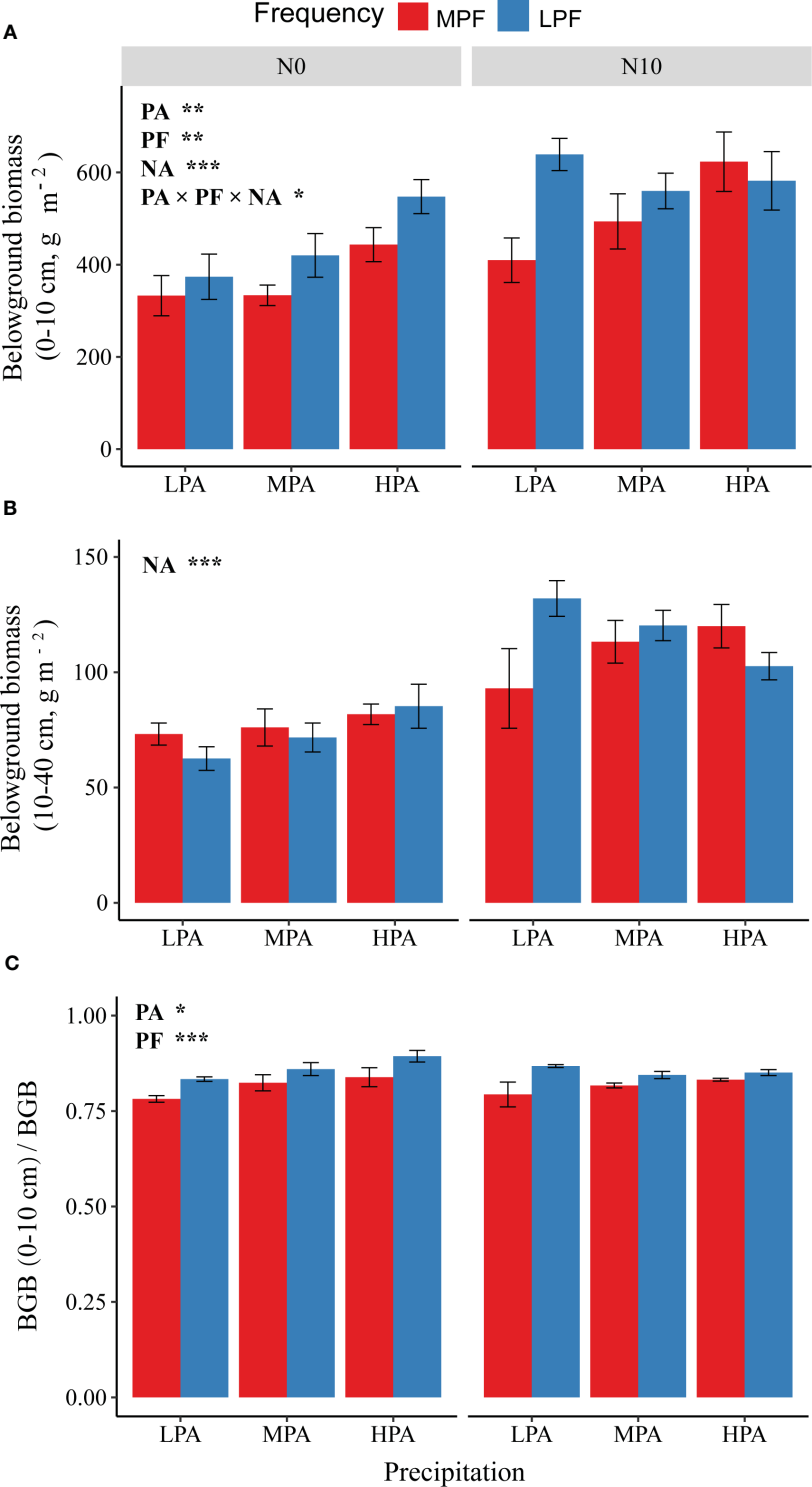
Effects of changes in precipitation pattern and nitrogen addition on belowground biomass at 0–10-cm **(A)** and 10–40-cm **(B)** soil depths, as well as the ratio of 0–10-cm BGB to total BGB **(C)**. The imposed treatments: precipitation amount (PA), low precipitation amount (LPA), medium precipitation amount (MPA), high precipitation amount (HPA), precipitation frequency (PF), low precipitation frequency (LPF), medium precipitation frequency (MPF), nitrogen addition (NA), nitrogen addition of 0 g N m^−2^ year^−1^ (N0), and nitrogen addition of 10 g N m^−2^ year^−1^ (N10). Data are presented as mean value ± 1 SE. Asterisks indicate the main treatment effects (*p < 0.05; **p < 0.01; ***p < 0.001). BGB, belowground biomass.

There were significant effects of the PA, PF, and NA treatments, as well as interactions, among these three treatments on BGB (0–10 cm) (**Figure 2A**). For BGB (10–40 cm), we only detected significant effects of the NA treatment (**Figure 2B**). There were significant effects of the PA and PF treatments on BGB (0–10 cm)/BGB; BGB (0–10 cm)/BGB increased with the increase in precipitation amount and reduction in precipitation frequency (**Figure 2C**).

### 3.4 Contribution of dominant species to total aboveground plant biomass

**Figure 3:**
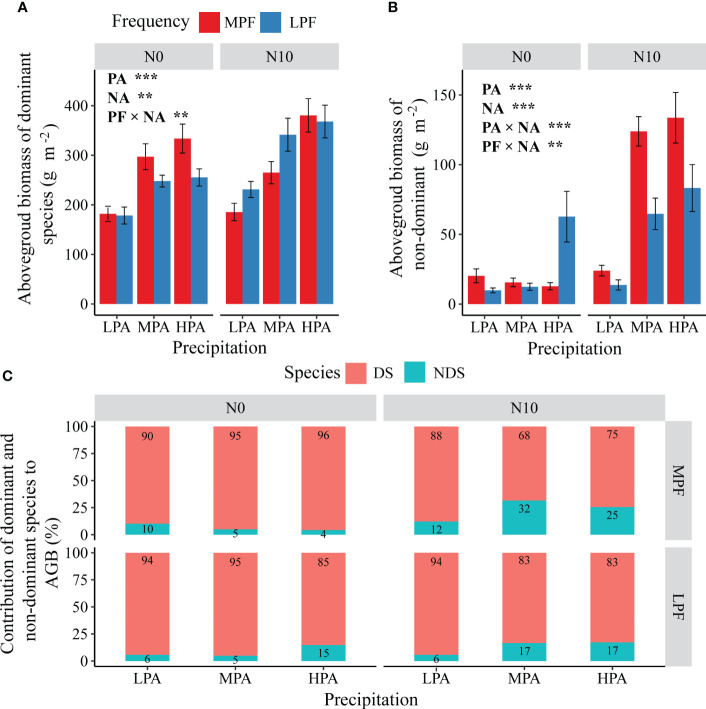
Effects of changes in precipitation pattern and nitrogen addition on aboveground biomass of dominant **(A)** and non-dominant species **(B)** and the contribution of dominant and non-dominant species to aboveground biomass **(C)**. The imposed treatments: precipitation amount (PA), low precipitation amount (LPA), medium precipitation amount (MPA), high precipitation amount (HPA), precipitation frequency (PF), low precipitation frequency (LPF), medium precipitation frequency (MPF), nitrogen addition (NA), nitrogen addition of 0 g N m^−2^ year^−1^ (N0), and nitrogen addition of 10 g N m^−2^ year^−1^ (N10). Data are presented as mean value ± 1 SE. Asterisks indicate the main treatment effects (**p < 0.01; ***p < 0.001).

**Figure 4:**
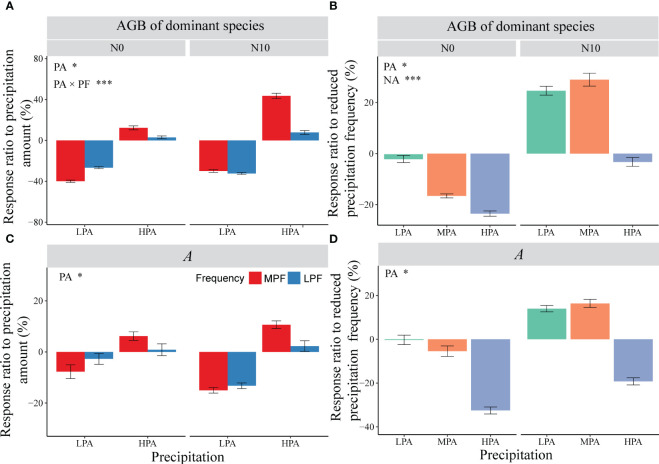
Effects of nitrogen addition on the response ratios of aboveground biomass of dominant species **(A, C)** and leaf carbon assimilation rate *(A)* **(B, D)** to the manipulated precipitation amount and frequency. The imposed treatments: precipitation amount (PA), low precipitation amount (LPA), medium precipitation amount (MPA), high precipitation amount (HPA), precipitation frequency (PF), low precipitation frequency (LPF), medium precipitation frequency (MPF), nitrogen addition (NA), nitrogen addition of 0 g N m^−2^ year^−1^ (N0), and nitrogen addition of 10 g N m^−2^ year^−1^ (N10). Data are presented as mean value ± 1 SE. Asterisks indicate the main treatment effects (*p < 0.05; ***p < 0.001).

For the dominant species (*L. chinensis*), we found significant effects of PA and NA, as well as interactions between PF and NA on AGB (**Figure 3A**). In general, nitrogen addition and an increase in precipitation amount resulted in an increase in the AGB of *L. chinensis*. Without nitrogen addition, the LPF treatment caused a decline (compared to MPF) in the AGB of *L. chinensis*; however, in the nitrogen addition mesocosms, the LPF treatment enhanced the AGB of *L. chinensis*, especially under the LPA and MPA conditions (**Figure 3A**). For the non-dominant species, there were significant effects of PA and PF, as well as interactions between treatments (PA × NA and PF × NA) on AGB (**Figure 3B**). The nitrogen addition treatment enhanced the AGB of the non-dominant plant species, but the LPF treatment caused a decline in the AGB of the non-dominant plant species in the fertilized mesocosms (**Figure 3C**). The experimental treatments had strong effects on the contribution of the non-dominant species to total AGB (**Figure 3C**); in general, nitrogen addition increased, but the LPF treatment reduced, the contribution of the non-dominant plant species (**Figure 3C**). For the response ratio to PA of the dominant species AGB, we detected significant effects of the PA treatment, as well as interactions between the PA and PF treatments. The LPF treatment reduced variation in precipitation amount-induced changes in the AGB of the dominant species (**Figure 4A**). The NA treatment tended to enhance the effects of reduced precipitation frequency on the dominant species AGB (**Figure 4B**).

### 3.5 Leaf gas exchange

**Figure 5:**
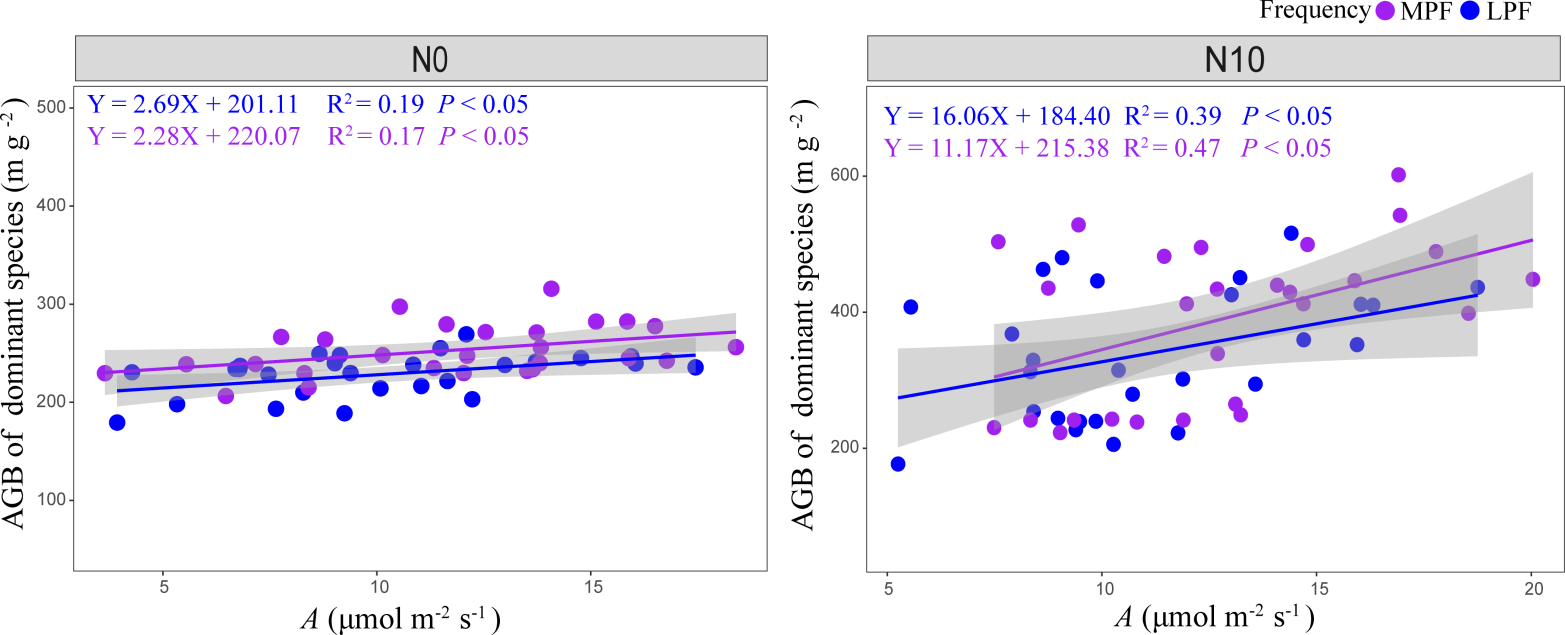
Dependence of aboveground biomass of dominant species on leaf carbon assimilation rate *(A)* under different precipitation frequencies (LPF, low precipitation frequency; MPF, medium precipitation frequency) and nitrogen (N0, 0 g N m^−2^ year^−1^; N10, 10 g N m^−2^ year^−1^) treatments. The equation, R^2^, and *p*-value of the relationships are provided.

We observed significant PA, PF, and NA treatment effects on the net assimilation rate of *L. chinensis* (**Supplementary Figure S6**). The response ratios of dominant species *A* to precipitation amount and precipitation frequency followed the same trend as the response ratios of dominant species AGB (**Figures 4C, D**). There were significant non-linear correlations between *A* and SWC (**Supplementary Figure S7**, *p* < 0.05). We detected strong positive dependence of aboveground biomass of dominant species on *A* for both medium and low precipitation frequency treatments (**Figure 5**, *p* < 0.05).

## 4 Discussion

### 4.1 Changes in precipitation patterns altered primary productivity and biomass allocation

Previous studies on the effects of changing precipitation patterns on grassland ecosystems have reported positive correlations (linear, exponential, and polynomial) between net primary productivity and annual precipitation (Bai et al., 2008; Hu et al., 2010; Kanniah et al., 2011; Guo et al., 2012). In this study, we also detected positive effects of enhancement in precipitation amount on leaf carbon assimilation rate and aboveground biomass (**Figure 1A**), which suggests that extra water addition alleviated plant drought stress (Luo et al., 2021; Yu et al., 2022). Moreover, we observed that less proportion of biomass was allocated to the belowground section under the high precipitation amount conditions (Dukes et al., 2005; Ren et al., 2017). According to the “optimal allocation hypothesis”, an increase in soil water availability allows the plant to allocate photosynthetic to organs that will enhance the obtaining of other limited resources, like light (Poorter et al., 2012). Similar results have been reported in other studies, where usually the scarcity (limitation) of resources causes plant traits to increase the rate of limiting resource acquisition (Eissenstat et al., 2000; Holdaway et al., 2011; Prieto et al., 2015).

In addition to precipitation amount, we found that changes in precipitation frequency also significantly affected biomass formation and allocation (**Figure 1**). As we expected, the low precipitation frequency treatment reduced aboveground biomass but enhanced root biomass and root-to-shoot ratio. However, the effects of reduced frequency depend on precipitation amount with greater impacts observed in the high precipitation amount mesocosms (**Figure 1B**). This phenomenon may have resulted because the soil moisture under the high precipitation amount and less precipitation frequency conditions (heavy rainfall events and longer rainfall intervals) was very unstable, exposing plants to flooding and severe drought, eventually limiting carbon assimilation (Zhang et al., 2017; Wang et al., 2019a). Waterlogging can harm plants through indirect effects, where waterlogging causes root hypoxia, affecting plant respiration, changing from normal autotrophic respiration to anaerobic respiration, resulting in insufficient supply of plant metabolites and energy (Pezeshki, 2001) and affecting the accumulation of aboveground plant parts. Moreover, the detected reduced precipitation frequency induced reduction in aboveground biomass may be related to changes in aboveground *vs.* belowground biomass allocation (more biomass was allocated to belowground; **Figure 1C**). In addition to the precipitation frequency reduction causing enhancement in belowground allocation, we also detected changes in root biomass allocation between the surface (0–10 cm) and deeper (10–40 cm) soil layers (**Figure 2**). However, contrary to our expectation, we detected an increase in 0–10-cm root biomass under the conditions of precipitation frequency reduction (**Figures 2A, C**). First, this phenomenon was likely a response to precipitation frequency reduction-induced shortage of nutrients in top soil; the plants enhanced root allocation in shallow soil to increase nutrient acquisition (Didiano et al., 2016; Yang et al., 2022). Second, the reduction in precipitation frequency is likely to result in a transitory waterlogging environment (especially when coupled with greater precipitation events), which may force the plant roots to grow toward the shallow layers to escape the anaerobic environment (Zhang et al., 2017; Wang et al., 2019b). Third, fine-textured soil (greater water holding capacity) may alleviate precipitation frequency reduction-induced severe shallow soil water scarcity (Araújo Santos et al., 2022; Gao et al., 2022), which contributes, to some extent, to the observed increase in 0–10-cm root ratio. We have to note that the lack of soil hydrological and biochemical data limited our ability to further unravel the underlying mechanisms of the observed mismatches between above- and belowground biomass responses to changes in precipitation regime. Therefore, future research should focus on understanding precipitation variation-induced comprehensive responses in soil hydrological and biochemical processes and their consequential effects on biomass formation and allocation.

### 4.2 Nitrogen addition regulated the effects of changes in precipitation patterns on plant biomass allocation

As we expected, N input significantly altered the effects of variation in precipitation patterns on biomass formation and allocation (**Figure 1**). The primary productivity of the studied grassland was N limited (Yang et al., 2011; Cui et al., 2021); therefore, the N addition significantly enhanced leaf carbon assimilation rate and biomass accumulation (**Figures 1A, C**). For the aboveground biomass, we detected significant interactions between changes in precipitation amount and N addition, which is in line with the results of a few recently published studies (Li et al., 2019b; Ma et al., 2020). In addition, the N addition treatment reduced the magnitude of precipitation frequency reduction-induced changes in AGB (**Figure 1B**). This phenomenon is likely related to the alleviation effects of nitrogen addition on low precipitation frequency-induced reduction in nitrogen availability. Greater precipitation events often result in nitrogen leaching and denitrification in surface soil, which will reduce nitrogen availability and impair plant growth. However, nitrogen addition may significantly alleviate the adverse effects of precipitation frequency reduction on nitrogen availability and eventually promote plant growth (Chen et al., 2021; Feng et al., 2023). The offsetting effects of N input on variation in precipitation frequency highlight the importance of considering interactions among global change factors when predicting their impacts on ecosystem processes and functions.

Despite N addition increased belowground biomass (**Figure 1C**), it apparently reduced the root-to-shoot ratio (**Figure 1E**). This is in line with the “optimal allocation hypothesis” and the results of many studies (Poorter et al., 2012; Eziz et al., 2017; Yang et al., 2018). Moreover, N addition increased root biomass at subsurface soil layers (10–40 cm) especially when soil water is limiting (**Figure 2B**). N addition-associated enhancement in carbon assimilation rate allows plants to form extra roots in subsurface soil layers, which not only helps the plants explore key resources but also likely enhances subsurface soil carbon storage.

### 4.3 Variation in precipitation pattern and nitrogen addition changed the dominancy of *L. chinensis*

**Figure 6:**
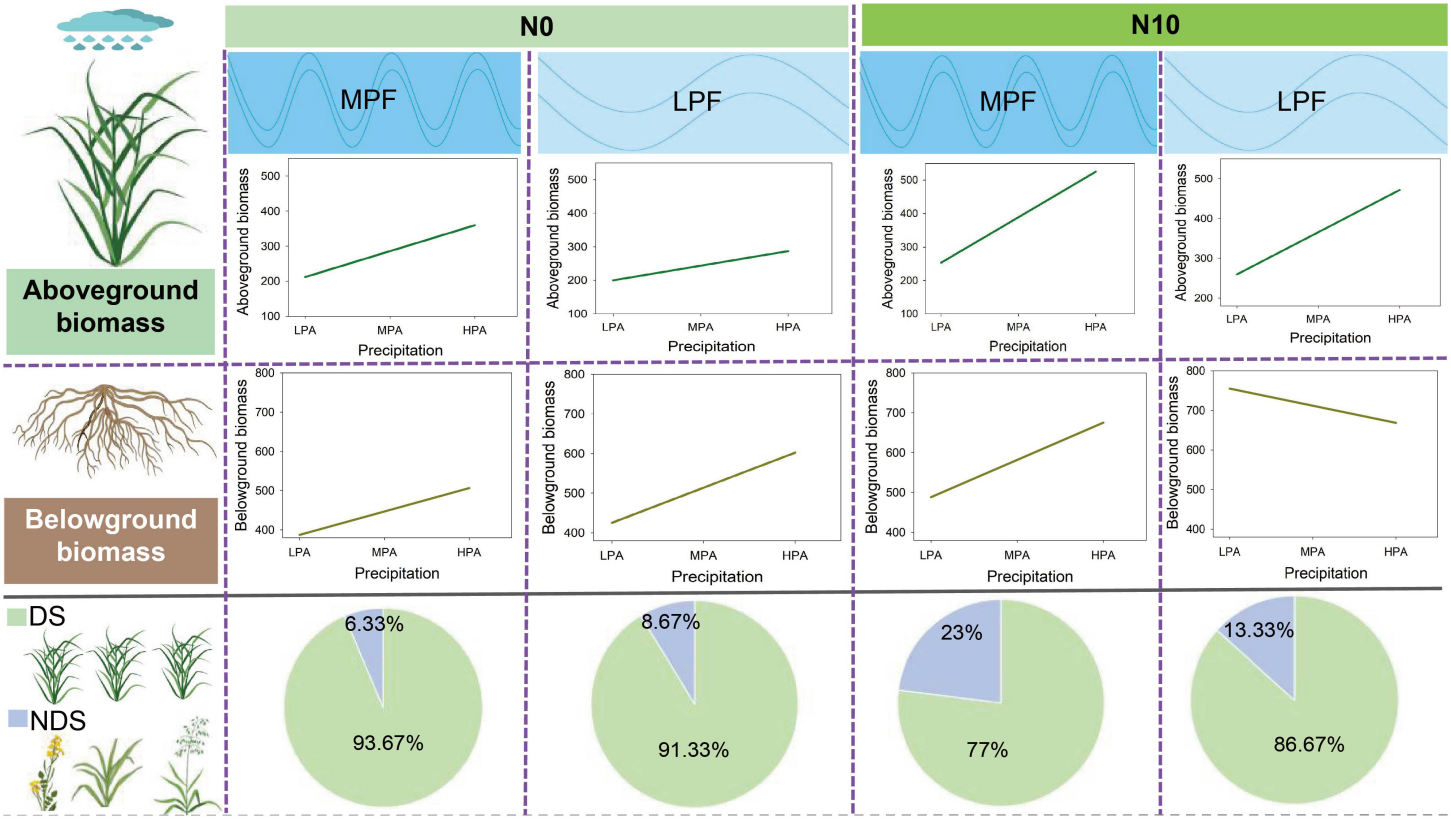
The regulating effects of precipitation frequency (LPF, low precipitation frequency; MPF, medium precipitation frequency) and nitrogen addition (N0, 0 g N m^−2^ year^−1^; N10, 10 g N m^−2^ year^−1^) on the responses of aboveground biomass and belowground biomass to changes in precipitation amount, and the contribution of dominant species (DS) *vs.* non-dominant species (NDS) to aboveground biomass.

Precipitation variability has great potential to alter vegetation composition and ecosystem functions, especially in water-limited ecosystems, such as grasslands (Bai et al., 2021; Guo et al., 2023). In this study, we found that the AGB of the dominant (*L. chinensis*) and non-dominant (*P. australis*, *K. integrifolia*, *C. virgata*, etc.) species increased with the increasing precipitation amount and nitrogen addition (**Figure 3**). However, the magnitude of enhancement was different between the dominant and non-dominant species. For example, the nitrogen addition treatment had much stronger enhancement effects on the aboveground biomass of the non-dominant species, especially under moderate and high precipitation conditions. These phenomena can be explained by the classical competitor, stress/tolerator/ruderal (CSR) theory (Pierce et al., 2017), which is that fast-growing species can benefit more from increased environmental resources such as water and nitrogen resources (Ma et al., 2022). In line with our findings, changes in precipitation patterns were reported to be more favorable for the growth of *Poa crymophila* and *Kobresia humilis*, thus changing the species composition of alpine grasslands (Ni et al., 2023). Similarly, a 2-year water addition experiment resulted in the dominant species shift from *Stipa tianschanica* to *Artemisia capillaris* (Ma et al., 2020). Despite the greater adaptability of the dominant species (*L. chinensis*) in Songnen meadow, our findings suggest that nitrogen deposition-associated grassland nutrient inputs (especially when interacting with changes in precipitation pattern) are likely to reduce the dominancy of *L. chinensis* and alter vegetation composition and productivity (**Figure 6**).

## 5 Conclusions

Changes in precipitation patterns strongly altered biomass formation and allocation of grassland ecosystems by affecting the magnitude of soil water variation and carbon assimilation of dominant species, but aboveground and belowground biomass responses to precipitation regimes were mismatched. Nitrogen addition mediated the magnitude and even direction of the effects of changes in precipitation patterns on primary productivity and biomass allocation. Moreover, nitrogen addition enhanced the contribution of non-dominant plant species to aboveground biomass, but the precipitation frequency reduction had the potential to maintain the dominancy of *L. chinensis*. Our findings highlight the importance of considering multiple global change factors when predicting their effects on grassland vegetation composition and ecosystem functions.

## Data availability statement

The original contributions presented in the study are included in the article/**Supplementary Material**. Further inquiries can be directed to the corresponding author.

## Author contributions

JR: Conceptualization, Formal Analysis, Investigation, Visualization, Writing – original draft, Writing – review & editing. CW: Formal Analysis, Visualization, Writing – original draft. QW: Formal Analysis, Visualization, Writing – original draft. WZS: Formal Analysis, Writing – original draft. WS: Conceptualization, Funding acquisition, Supervision, Writing – original draft, Writing – review & editing.
