# Supplementary material for: Nitrogen addition regulates the effects of variation in precipitation patterns on plant biomass formation and allocation in a Leymus chinensis grassland of northeast China
Source: Front Plant Sci. 2024 Jan 12;14:1323766. doi: 10.3389/fpls.2023.1323766 (PMC10810989; doi:10.3389/fpls.2023.1323766)
Supplement: Supplementary file 1 [file DataSheet_1.doc]

**Nitrogen addition regulates the effects of variation in precipitation pattern on plant biomass formation and allocation in a *Leymus*** ***chinensis* grassland of northeast China**

Jianli Ren^1, 2, 3^, Chengliang Wang^1^, Qiaoxin Wang^1^, Wenzheng Song^1^ , Wei Sun^1, 4, *^

*1 Institute of Grassland Science, Key Laboratory of Vegetation Ecology of the Ministry of Education, Jilin Songnen Grassland Ecosystem National Observation and Research Station, Northeast Normal University, Changchun, Jilin 130024, China*

*2* *School of Resources and Environment, Yili Normal University, Yining,* *Xinjiang 835000, China*

*3 Institute of Resources and Ecology, Yili Normal University, Yining, Xinjiang 835000, China*

*4 State Environmental Protection Key Laboratory of Wetland Ecology and Vegetation Restoration, Northeast Normal University, Changchun, Jilin 130024, China*

**Correspondence:**

Corresponding Author: Wei Sun

1. mail address: sunwei@nenu.edu.cn (Wei Sun)


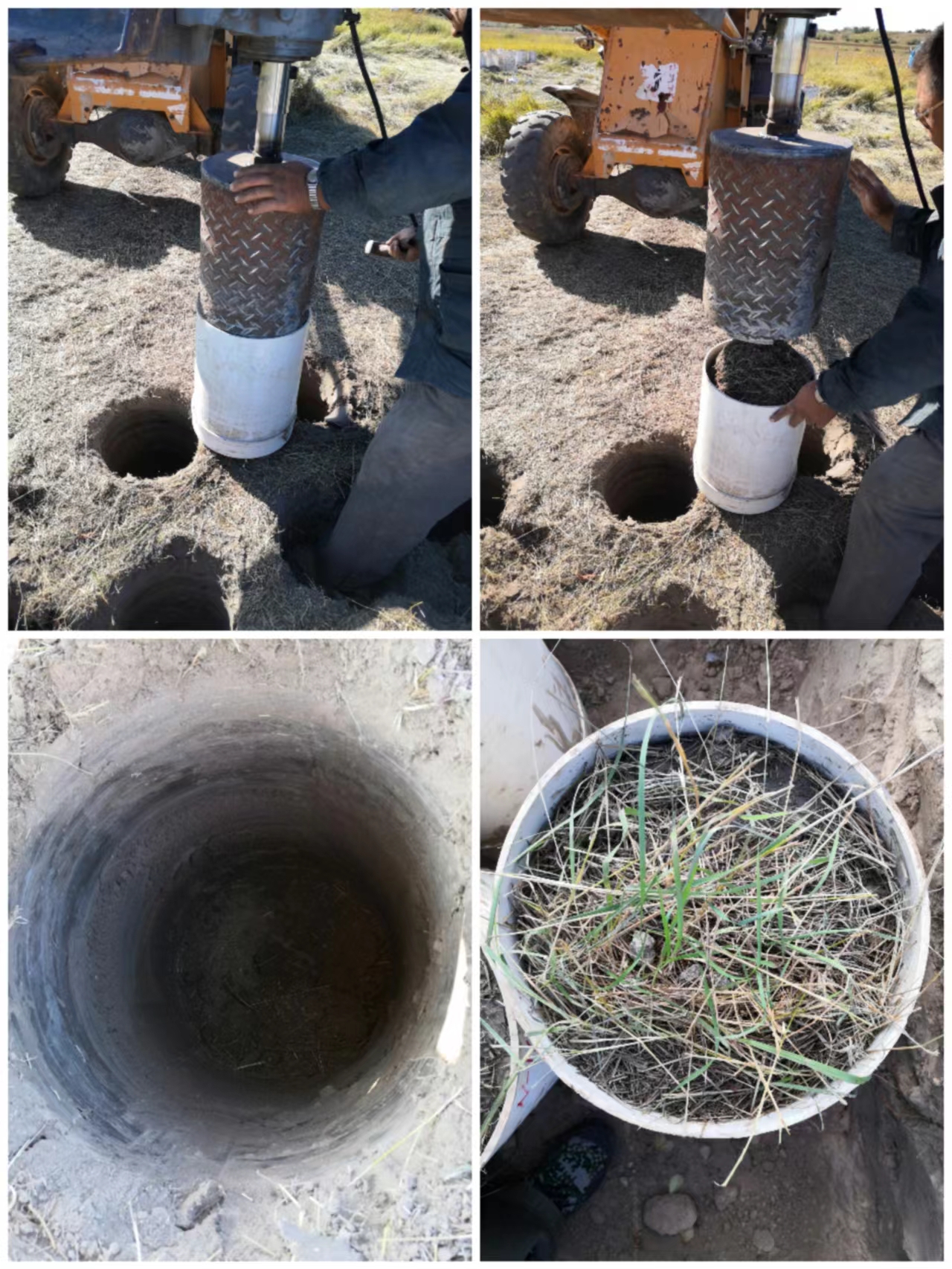


Figure S1. Field photos of soil column collection.


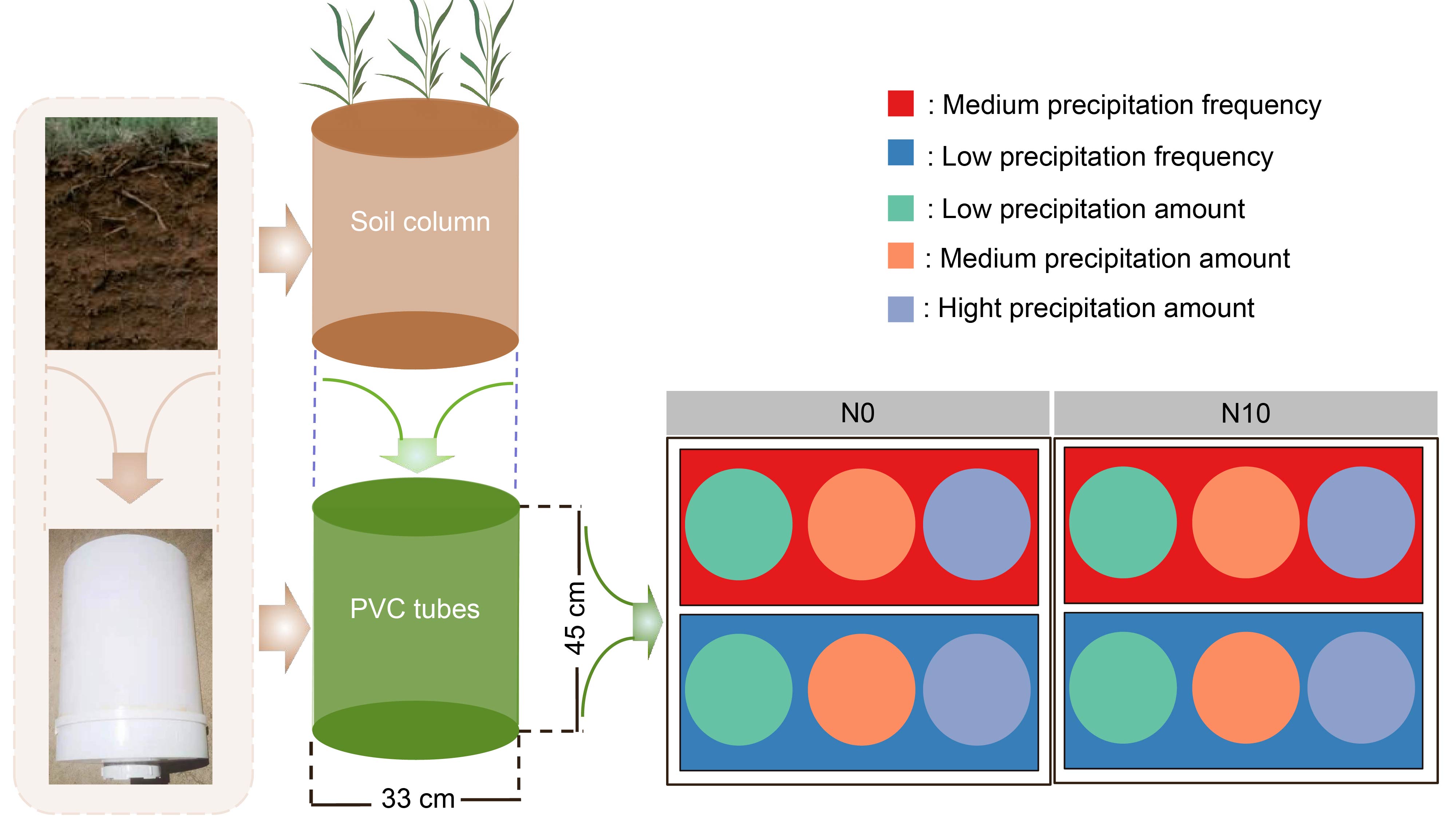


Figure S2. Experimental treatment and experimental layout.


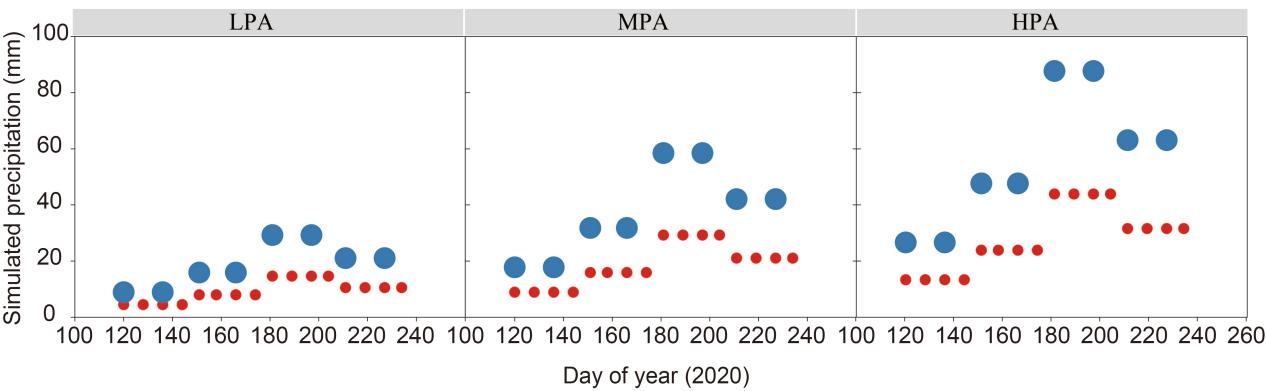


Figure S3. Timing and size of the manipulated precipitation events for the three precipitation amount and two precipitation frequency treatments.


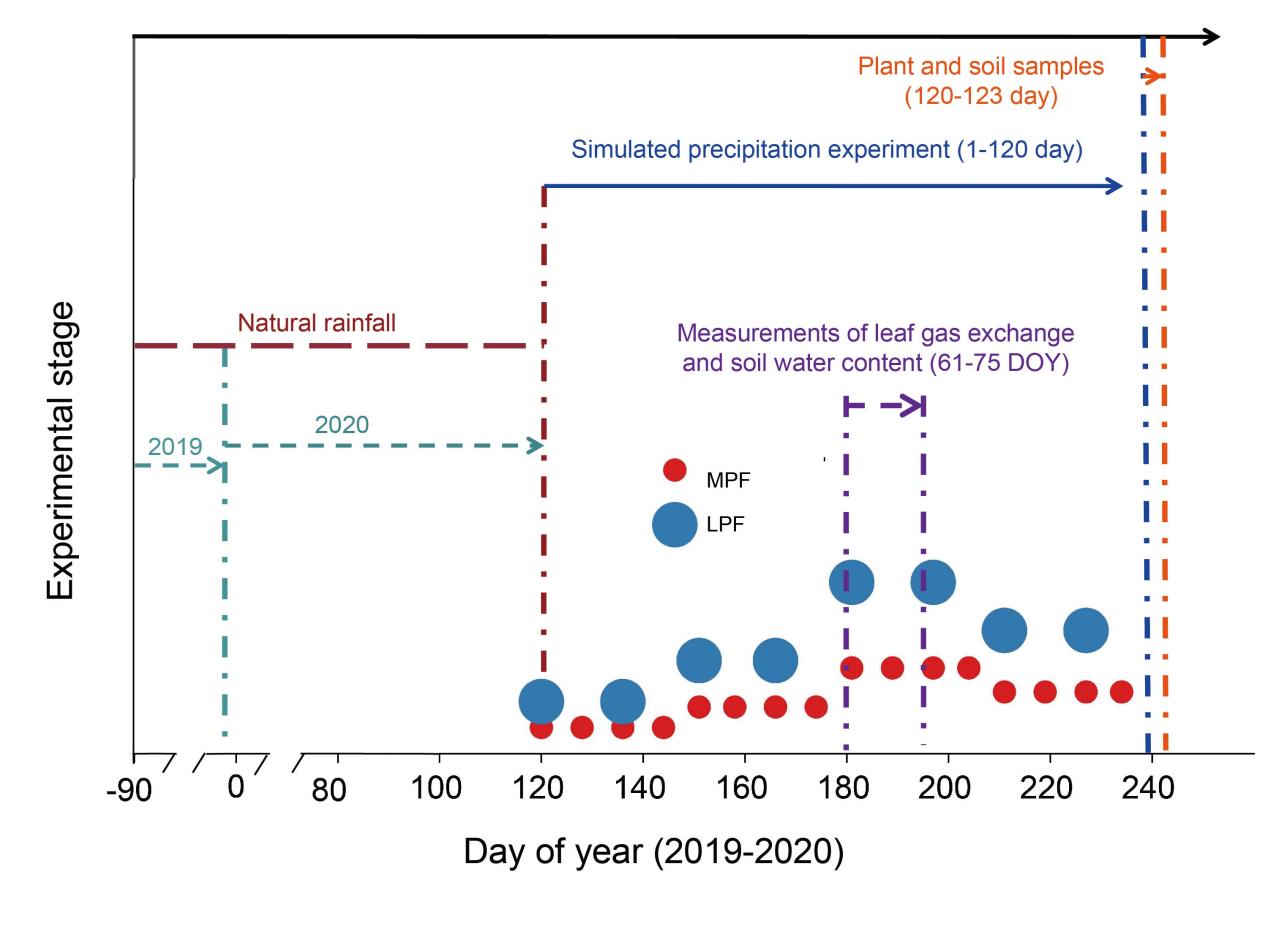


Figure S4. A diagram showing the different stages of the manipulative experiment and their corresponding timing and duration.


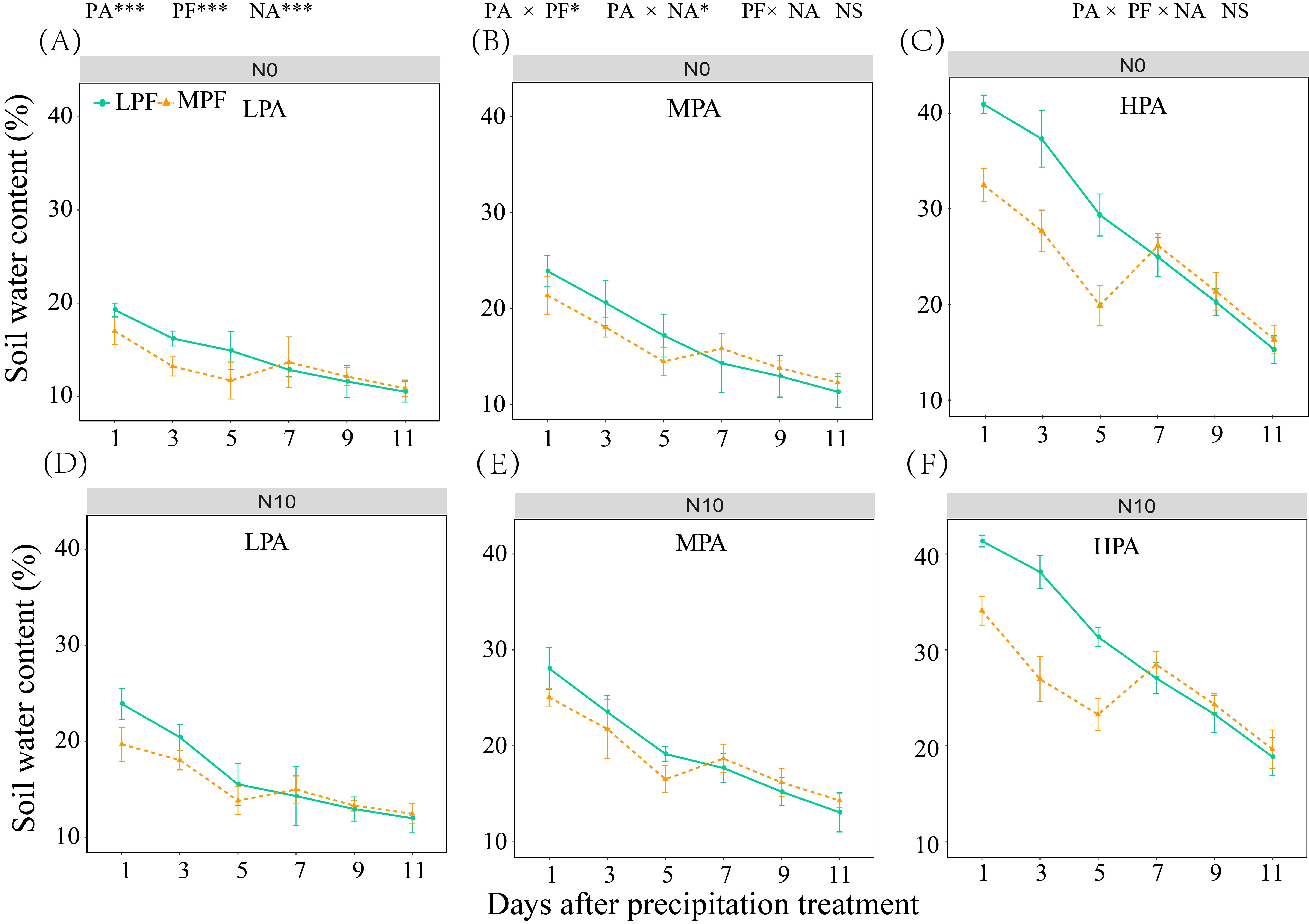


Figure S5. Effects of the precipitation pattern and nitrogen addition treatments on soil water content (0-10 cm). The imposed treatments including: precipitation amount (PA), low precipitation amount (LPA), medium precipitation amount (MPA), high precipitation amount (HPA), precipitation frequency (PF), low precipitation frequency (LPF), medium precipitation frequency (MPF), nitrogen addition (NA), nitrogen addition is 0 g N m^-2^ yr^-1^ (N0), nitrogen addition is 10 g N m^-2^ yr^-1^ (N10). Data are presented as mean value ± 1 SE.


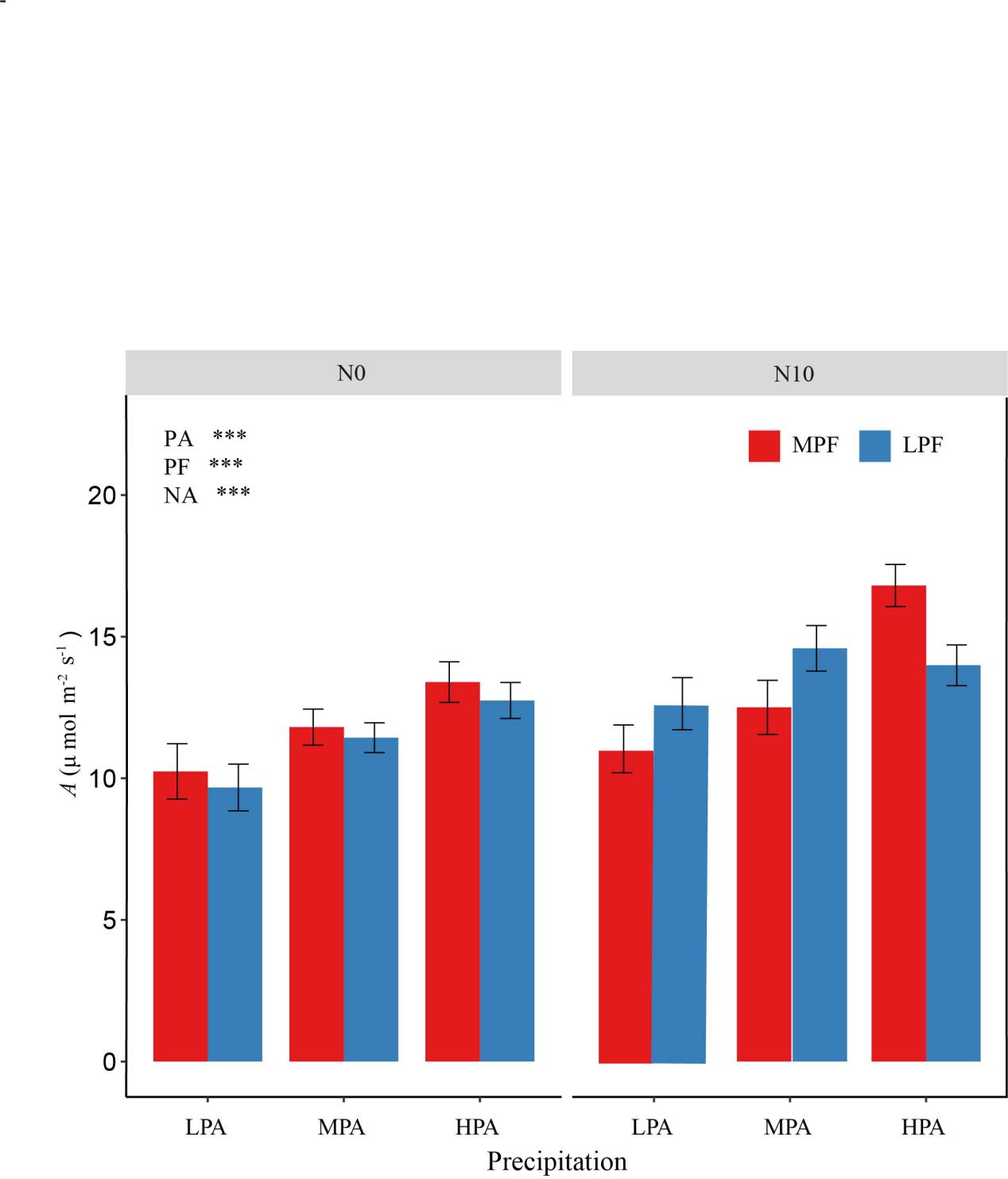


Figure S6. Effects of the precipitation pattern and nitrogen addition treatments on leaf carbon assimilation rate (*A*). The imposed treatments including: precipitation amount (PA), low precipitation amount (LPA), medium precipitation amount (MPA), high precipitation amount (HPA), precipitation frequency (PF), low precipitation frequency (LPF), medium precipitation frequency (MPF), nitrogen addition (NA), nitrogen addition is 0 g N m^-2^ yr^-1^ (N0), nitrogen addition is 10 g N m^-2^ yr^-1^ (N10). Data are presented as mean value ± 1 SE.


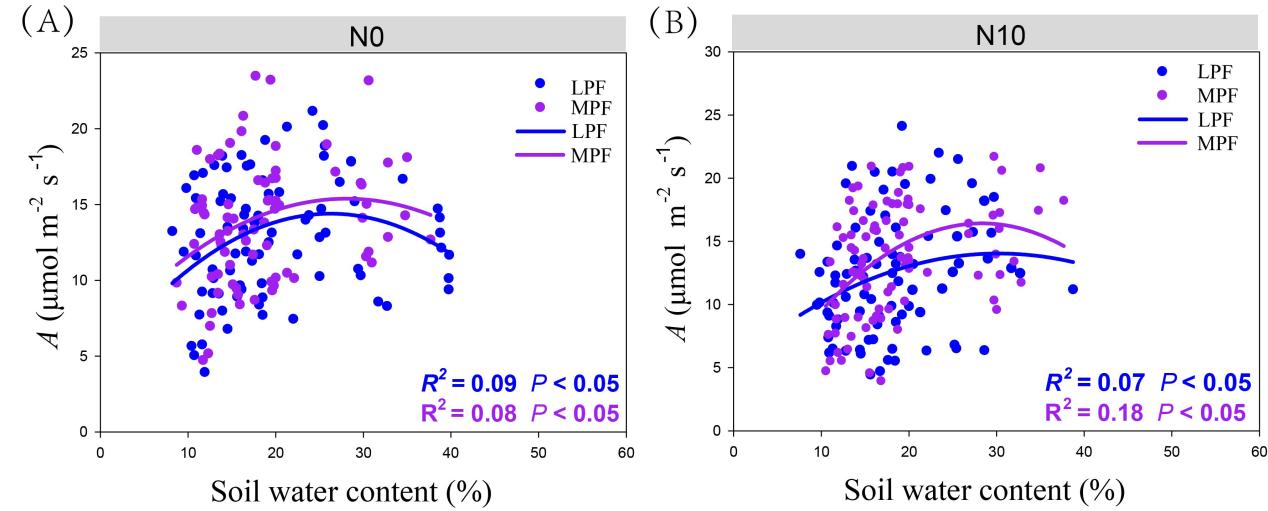


Figure S7. Interrelationships between soil water content and leaf carbon assimilation rate (*A*) of dominant species under different precipitation patterns (LPF: low precipitation frequency; MPF: medium precipitation frequency) and nitrogen addition (N0: 0 g N m^-2^ yr^-1^; N10: 10 g N m^-2^ yr^-1^) treatments.
